# Wearable IMMU-Based Relative Position Estimation between Body Segments via Time-Varying Segment-to-Joint Vectors

**DOI:** 10.3390/s22062149

**Published:** 2022-03-10

**Authors:** Chang June Lee, Jung Keun Lee

**Affiliations:** 1 Inertial Motion Capture Lab, Department of Mechanical Engineering, Hankyong National University, Anseong 17579, Korea; 2019563205@hknu.ac.kr; 2 Inertial Motion Capture Lab, School of ICT, Robotics & Mechanical Engineering, Hankyong National University, Anseong 17579, Korea

**Keywords:** relative position estimation, human motion capture, soft tissue artifacts, segment-to-joint vector, inertial and magnetic measurement units

## Abstract

In biomechanics, estimating the relative position between two body segments using inertial and magnetic measurement units (IMMUs) is important in that it enables the capture of human motion in unconstrained environments. The relative position can be estimated using the segment orientation and segment-to-joint center (S2J) vectors where the S2J vectors are predetermined as constants under the assumption of rigid body segments. However, human body segments are not rigid bodies because they are easily affected by soft tissue artifacts (STAs). Therefore, the use of the constant S2J vectors is one of the most critical factors for the inaccurate estimation of relative position. To deal with this issue, this paper proposes a method of determining time-varying S2J vectors to reflect the deformation of the S2J vectors and thus to increase the estimation accuracy, in IMMU-based relative position estimation. For the proposed method, first, reference S2J vectors for learning needed to be collected. A regression method derived a function outputting S2J vectors based on specific physical quantities that were highly correlated with the deformation of S2J vectors. Subsequently, time-varying S2J vectors were determined from the derived function. The validation results showed that, in terms of the averaged root mean squared errors of four tests performed by three subjects, the proposed method (15.08 mm) provided a higher estimation accuracy than the conventional method using constant vectors (31.32 mm). This indicates the proposed method may effectively compensate for the effects of STAs and ultimately estimate more accurate relative positions. By providing STA-compensated relative positions between segments, the proposed method applied in a wearable motion tracking system can be useful in rehabilitation or sports sciences.

## 1. Introduction

Recent advances in wearable sensing technology have enabled the continuous monitoring of physical activities in various applications such as rehabilitation, sports science, and medical care. The wearable inertial and magnetic measurement unit (IMMU) is a low-cost, small-sized wearable motion sensor that can detect human motion with high precision in unconstrained environments. In addition, it allows body posture [1,2,3,4,5] or position [6,7,8,9,10] information to be estimated from sensor signals. Owing to these advantages, IMMUs have been effectively used to capture and analyze human motion in various environments (e.g., outdoors). For example, studies have been conducted to analyze human gait [11,12,13,14] or estimate the position of pedestrians [15,16,17,18] using IMMUs attached to lower body segments.

In IMMU-based motion capture technologies, the three-dimensional (3D) relative position between body segments is an important physical quantity that provides fundamental kinematic information [19,20,21,22,23]. The relative position can be estimated using the segment orientations and segment-to-joint center (S2J) vectors, as the body segments are connected in a chain through a joint [17,18,19,20,21,22,23]. The segment orientation can be estimated using a sensor fusion algorithm (e.g., the Kalman filter, KF) [1,2,3,4,5] from sensor signals, and S2J vectors can be determined using premeasured length information [22,23] or via calibration [24,25,26]. This method is performed based on the assumption that the body segments are rigid bodies connected by a mechanical spherical joint (e.g., ball-and-socket joint). As such, the S2J vector is regarded as a constant. Once the S2J vectors have been predetermined, the relative position is determined only by the orientation information. Therefore, the relative position is often regarded as a byproduct of orientation estimation.

**Figure 1:**
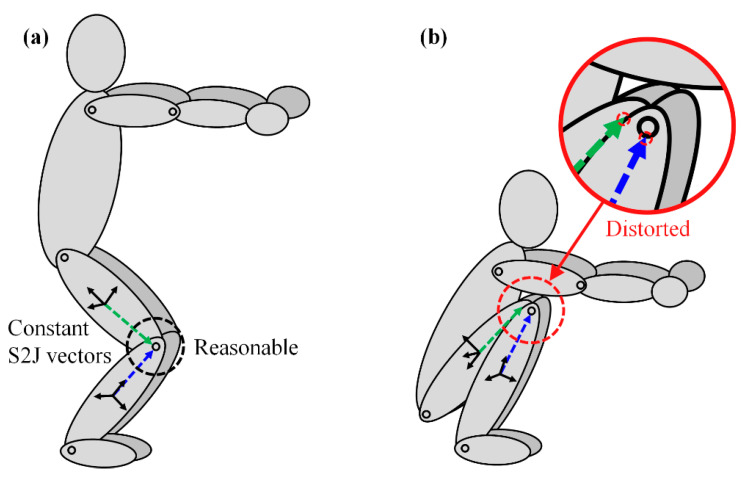
Segment-to-joint (S2J) vectors during a squatting motion: (**a**) slightly bent knee; (**b**) fully bent knee.

However, human body segments are not rigid bodies, as they are easily affected by the deformation and sliding of the skin over the underlying bone as well as muscular contractions. In addition, human body segments are connected by soft elements, such as muscles, tendons, and cartilage, unlike a mechanical joint. As such, the position of the joint center relative to the body segment is not fixed. These body deformations are known as soft tissue artifacts (STAs). In the case of performing a squat, for example, predetermined constant S2J vectors can be applied reasonably when the knee is slightly bent as shown in Figure 1a, but they will be distorted from the truth S2J vectors when the knee is fully bent as shown in Figure 1b. Therefore, the use of the constant S2J vectors is one of the most critical factors for the inaccurate estimation of relative position.

Due to the STA, the skin-attached noninvasive sensors have a movement that is separated from the skeleton to some extent, resulting in inaccurate estimations. For example, in camera-based optical motion capture, STAs cause differences between the skin-attached marker and anatomical landmark, resulting in errors when defining the anatomical frame or joint coordinate system from the marker. Therefore, studies have been conducted to compensate for or quantify the effects of STAs to improve the estimation accuracy of optical motion capture [27,28,29,30]. By contrast, IMMUs contain limited measurable information to compensate for STAs. Furthermore, in general, only one IMMU is used for one body segment in IMMU-based motion capture, while the STAs on one body segment appear as high-dimensional geometric transformations (e.g., translation, rotation, and change in size/shape). These make the effect of STAs on IMMU-based motion tracking difficult to consider. In this regard, there are only a few related studies as described below.

Frick and Rahmatalla [31] proposed a method for estimating the joint center position with respect to the sensor frame as a time-varying vector to consider the variation in the joint center due to STAs. The method proposed in [31] uses the equation of joint center acceleration as an optimization problem to determine the joint center for each time point. Instead of an optimization approach, García-de-Villa et al. [32] estimated the joint center in real time by designing an extended KF where the same constraint equation in [31] was used for the measurement step of the extended KF. These methods are disadvantageous in that estimations are difficult to perform in dynamic conditions because they are applicable only when the acceleration of the joint center is negligible. However, they are useful in that they quantify the variation in the joint center due to STAs. The authors of this paper, Lee and Lee [33], proposed a KF that estimates the S2J vectors for each segment as time-varying vectors to consider the variation in the joint center when estimating the relative position between two body segments. Although the method proposed in [33] compensated for the effects of STAs by applying a constraint of the joint center acceleration, uncertainty existed because the constraint is based on a mechanical spherical joint.

This paper proposes a method of determining time-varying S2J vectors to reflect the deformation of the S2J vectors and thus to increase the estimation accuracy, in IMMU-based relative position estimation. In terms of methodology, first, reference S2J vectors for learning needed to be collected. Then, a regression method derived a function outputting S2J vectors based on specific physical quantities that were highly correlated with the deformation of S2J vectors. Subsequently, time-varying S2J vectors were determined from the derived function. This study shows that whereas the method of using the constant vectors is affected by body deformation, the proposed method of using time-varying vectors can effectively compensate for the effect of deformation.

## 2. Materials and Methods

### 2.1. Relative Position Estimation Based on Orientation and S2J Vectors

**Figure 2:**
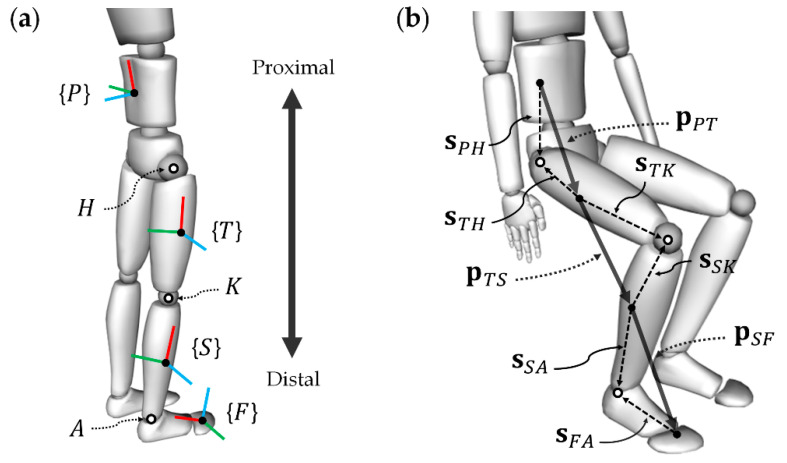
Lower body model: (**a**) segment frames and joints; (**b**) segment-to-joint vectors and relative position vectors.

This study pertains to relative position estimation in the region encompassing the pelvis, thigh, shank, and foot, where the coordinates of these segments are denoted as {*P*}, {*T*}, {*S*}, and {*F*}, respectively. It is assumed that the coordinate system of the sensor attached to each body segment is the same as that of the corresponding segment. These body segments are connected in series by the hip, knee, and ankle joints, and these joints are denoted as *H*, *K*, and *A*, respectively. Figure 2a shows the coordinate systems used for the segments and joint locations in the lower body model.

The orientation of a segment can be expressed using the direction cosine matrix. For example, the relative orientation of the thigh with respect to the pelvis is RTP=RPITRTI, where RPI and RTI are the orientations of the pelvis (*P*) and thigh (*T*) with respect to the fixed inertial frame (*I*), respectively, and the right superscript *T* indicates that the matrix is transposed. The orientation of each segment can be estimated using the sensor signals of the IMMU and the sensor fusion algorithm. The coordinate transformation of a 3 × 1 vector **x** using the relative orientation of two body segments is as follows:

(1) xP=RTPxT,

where the left superscripts indicate that the vectors are observed in the coordinate of each segment.

Lower body segments are connected sequentially through joints and can be categorized into proximal (*p*) and distal (*d*) segments based on a specific joint *J* (i.e., *H*, *K*, and *A*∈J). For the hip joint as an example, *p* and *d* correspond to {*P*} and {*T*}, respectively (see Figure 1). When a joint connecting two adjacent segments is modeled as a spherical joint, the relative position from *p* to *d* is determined as follows:

(2) pPpd=sppJ−RdpsddJ,

where sppJ and sddJ are the vectors from *p* and *d* to *J*, respectively, i.e., the S2J vectors. In general, S2J vectors are determined to be constant based on calibration. Hence, once the S2J vectors have been predetermined, the relative position is determined solely based on the orientation information, and this approach allows the relative positions pPPT, pTTS, and pSSF to be determined.

In addition, the relative position between nonadjacent segments can be determined through the kinematic propagation in a chain of joints. The relative position from the pelvis to the foot, corresponding to the proximal and distal ends of the lower body, was determined using the relative position between adjacent segments, as follows:

(3) pPPF=pPPT+RTPpTTS+RTPRSTpSSF

### 2.2. Determination of Time-Varying S2J Vectors

In the relative position estimation method introduced above, S2J vectors are regarded as constants because the body segments are assumed to be rigid, and joints are modeled as spherical joints. However, as mentioned earlier, because human body segments are affected by STAs, the use of constant S2J vectors in Equation (2) resulted in inaccurate relative position estimation. Hence, the proposed method compensated for the effects of STAs by determining the time-varying S2J vectors and applied them to the relative position estimation. In the proposed method, it is assumed that each joint exhibits specific variables that were highly correlated with the deformation of the S2J vector owing to STAs (i.e., STA-related variables). Based on this assumption, the proposed method estimated an equation for S2J vectors as a function of STA-related variables through regression, and then determined the S2J vectors as time-varying vectors from the estimated function.

The proposed method was divided into two depending on the number of STA-related variables: one approach used a single variable as an input of the function, and the other used two variables. In the first approach, which used a single STA-related variable $\left(x_{J}\right)$, the correlation between $x_{J}$ and S2J vectors (yJ=[sppJTsddJT]T) is modeled as $y_{J}=f(x_{J})$, where the S2J vector estimator $f^{*}(x_{J,t})$ is derived via kernel regression [34]. By contrast, the second approach, which used two variables ($x_{J,1,t}$ and $x_{J,2,t}$), derived the function of the S2J vector in the form of $f^{*}(x_{J,1,t},x_{J,2,t})$ via multivariate regression [35].

In this study, it is assumed that the joint angle is highly correlated with the deformation of the S2J vector. In particular, the rotational motion, which is governed by the rotation of the joint, affects the body deformation. Therefore, the proposed method used the joint angle as an STA-related variable. First, the flexion angle of each joint is used as an STA-related variable for $f^{*}(x_{J,t})$ because all three joints in the lower body (*H*, *K*, and *A*) experience the widest range of rotations in the sagittal plane. For example, movements such as squats and sit–to–stand are performed mainly with the flexion/extension of hip, knee, and ankle. Second, the flexion angles of the two joints are used as STA-related variables for $f^{*}(x_{J,1,t},x_{J,2,t})$, under the assumption that body deformation is also affected by the rotation of other joints close to the proximal segment. For example, in the case of S2J vectors on the ankle, i.e., sSSA and sFFA, the flexion angles of the ankle and knee are used. As an exception, the hip joint is located in the proximal region of the lower body. Therefore, hip flexion and abduction angles are used as STA-related variables.

### 2.3. Estimation of Reference S2J Vectors for Learning

The regression method derived a function outputting S2J vectors based on STA-related feature data. Therefore, learning data comprising the reference S2J vector should be prepared. However, owing to insufficient information regarding the joint center position, the truth reference S2J vector is difficult to determine. Therefore, this study proposes a KF estimating S2J vectors s^ppJ and s^ddJ where the vectors satisfy the reference relative position.

The basic structure of the KF can be defined as a process model $x_{t}=\Phi_{t-1}x_{t-1}+w_{t-1}$ and a measurement model $z_{t}=H_{t}x_{t}+v_{t}$, where **x** is a state vector, **z** is a measurement vector, Φ is a state transition matrix, **H** is an observation matrix, and **w** and **v** are the process and measurement noise, respectively. Because the purpose of the KF is to estimate the S2J vectors for the learning data, the state vector is defined as xt=[s^ppJ,tTs^ddJ,tT]T.

Because the behavior of the S2J vectors as a result of STAs is highly complex, it is difficult to predict the S2J vectors as time progresses. Therefore, assuming that the deformation of S2J vectors during a sampling time Δt is insignificant, the process model can be defined as follows:

(4) [s^ppJ,ts^ddJ,t]=[I3O3O3I3][s^ppJ,t−1s^ddJ,t−1]+wt−1

**Figure 3:**
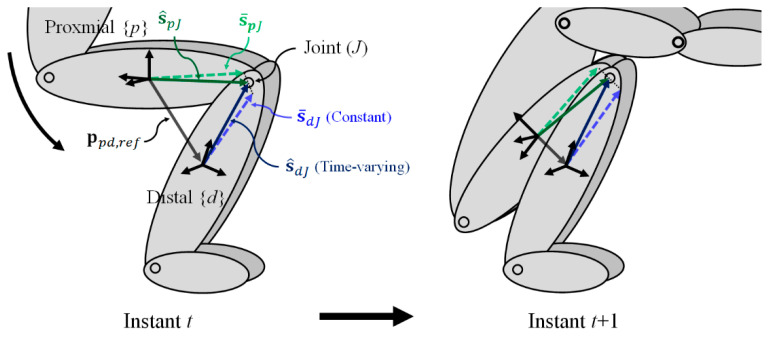
S2J vectors that satisfy truth reference relative position.

While the constant S2J vector determined via optimization techniques does not reflect deformation caused by STAs, it can still provide the approximate position information of a joint for a certain segment. In this regard, constant vectors are used as measurements in the KF. In the proximal region, constant S2J vector s¯ppJ is modeled as s¯ppJ=s^ppJ,t+δpJ, where $\bar{s}$ indicates that the S2J vector is constant, and δ is the deformation of the S2J vector. In addition, because the S2J vector must satisfy Equation (2) when the truth references of relative orientation Rdpref and position pppd,ref measured via motion capture are applied, the following measurement equation is obtained:

(5) pPpd,ref=sppJ−RdprefsddJ,

therefore, the measurement model can be defined as follows:

(6) [s¯ppJs¯ddJpppd,ref,t]=[I3O3O3I3I3−Rdpref,t][s^ppJ,ts^ddJ,t]+[δpJδdJ03×1],

where $\delta_{pJ}$ and $\delta_{dJ}$ determine the fusion weights of s¯ppJ and s¯ddJ, respectively, and $0_{3\times 1}$ indicates that s^ppJ,t and s^ddJ,t must satisfy the truth relative position, as shown in Figure 3.

### 2.4. Validation

**Figure 4:**
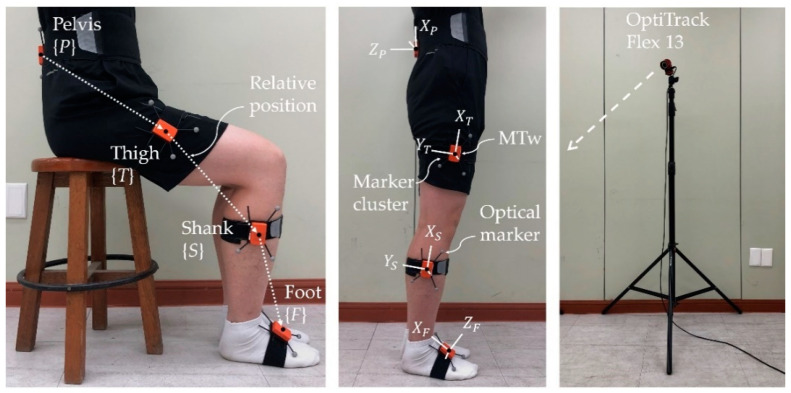
Experimental setup: (**left**) Definition of lower body segments and relative position. (**middle**) One MTw fixed to marker cluster plate is attached to each body segment. (**right**) OptiTrack Flex 13 is used to track optical markers.

To validate the proposed method, we used an MTw IMMU (Xsens Technologies B.V., Enschede, The Netherlands), which included a gyroscope, an accelerometer, and a magnetometer. Each IMMU was attached to the back of the pelvis, to the sides of the thigh and shank, and to the foot using a Velcro band, in the same direction as the coordinate system shown in Figure 1: Red corresponds to the *x*-axis, green to the *y*-axis, and blue to the *z*-axis. The truth references of the orientation and position for the learning and performance verification of the proposed method were provided by the OptiTrack Flex 13 camera motion capture system (NaturalPoint, Inc., Corvallis, OR, USA). The sampling rate for both systems was set to 100 Hz. In addition, the IMMU was fixed to a marker cluster plate with three reflective markers forming a plane, which enabled to calculate the truth reference orientation, as shown in Figure 4.

Three healthy male subjects (age: 26.7 ± 0.6 years; mass: 80.3 ± 9.5 kg; height: 1.76 ± 0.05 m) participated in the validation tests. The constant S2J vectors for each subject were determined using the least squares method proposed in [24] and are listed in Table 1.

**Figure 5:**
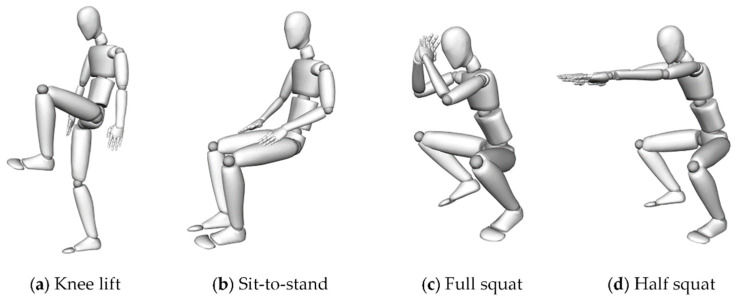
Experimental motions of (**a**) Test 1, (**b**) Test 2, (**c**) Test 3, and (**d**) Test 4.

In the validation experiment, all subjects performed knee lift (Test 1), sit–to–stand (Test 2), full squat (Test 3), and half squat (Test 4) motions, as illustrated in Figure 5. These test movements were chosen as they involve body deformations due to the bending of the lower limb joints but have different degrees of deformations each other. Each test was performed for five trials, in which each trial lasted 120 s. For each test, data from the first trial were used to construct learning data for regression, whereas remaining data were used to validate the proposed method.

For each test, the 3D relative position was estimated using four different methods, as follows: Method 1 (M1) is a conventional method that applies constant S2J vectors (s¯ppJ and s¯ddJ), as well as the truth reference orientation (Rdpref) to Equation (2). The estimation error of M1 arises from only the deformation of the S2J vectors based on the body deformation, which may be considered as the effect of STAs to be compensated. Both methods 2 (M2) and 3 (M3) are the proposed method. However, M2 used the single variable, and M3 used two variables to determine the time-varying S2J vectors. Furthermore, M2 and M3 used univariate regression [34] and multivariate regression [35], respectively, to derive the function for the S2J vector in the form of $f^{*}(x_{J,t})$ and $f^{*}(x_{J,1,t},x_{J,2,t})$, respectively. These methods applied the time-varying S2J vectors determined via each approach and Rdpref to Equation (2). Method 4 (M4) is the same approach as M3, where the estimated relative orientation (Rdpest=RpIestTRdIest) is used instead of Rdpref to determine the relative position and extract the joint angle. Orientations RpIest and RdIest used in M4 were estimated using the IMMU-based orientation estimation algorithm having a model-based disturbance compensation mechanism [5]. The estimation accuracies of the four methods were evaluated using the root mean squared errors (RMSEs) of the relative position estimation. To compare the estimation accuracy efficiently, the RMSEs from the four trials were averaged for each test.

## 3. Results and Discussion

**Figure 6:**
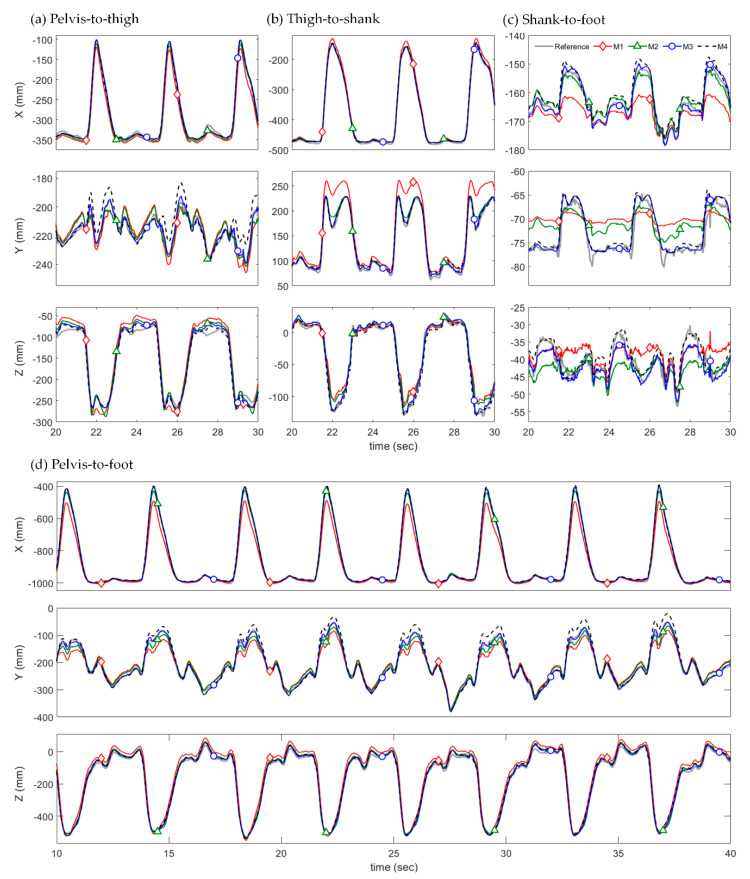
Estimated 3D relative positions from M1 (*red diamond*), M2 (*green triangle*), M3 (*blue circle*) and M4 (*black dashed*) with the truth reference (*gray solid line*) of Test 1 (knee lift) from Subject 1.

**Figure 7:**
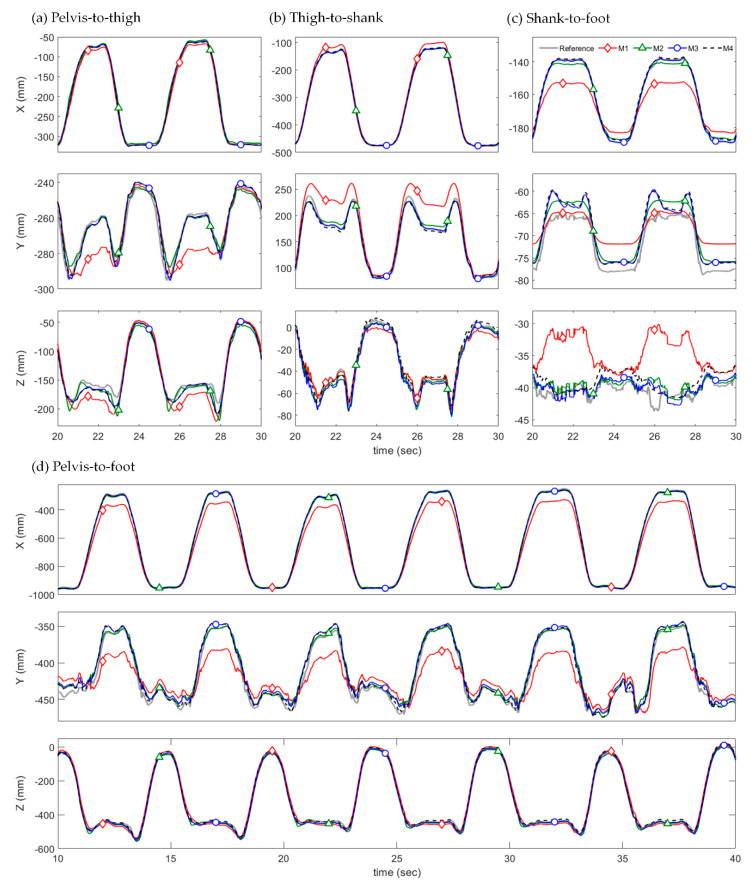
Estimated 3D relative positions from M1 (*red diamond*), M2 (*green triangle*), M3 (*blue circle*) and M4 (*black dashed*) with the truth reference (*gray solid line*) of Test 3 (full squat) from Subject 1.

Table 2 lists the averaged RMSEs of the relative position estimated using the four different methods. Table 3 lists the averaged RMSEs of the estimated 3D joint angles applied to M4. Figure 6 and Figure 7 show the 3D relative position estimation results for pelvis–to–thigh, thigh–to–shank, shank–to–foot, and pelvis–to–foot of Tests 1 and 3 from Subject 1.

In most tests, the proposed methods (M2–M4) showed smaller RMSEs of relative positions than the conventional method (M1), which used constant S2J vectors. This was particularly clear in the results for pelvis–to–foot (pPPF) as the pelvis–to–foot vector was determined by incorporating the three relative positions of adjacent segments (pPPT, pTTS, and pSSF) into Equation (3). For Tests 1–4 of three subjects, for example, the averaged RMSE of M1 was 25.05 mm, whereas those of M2, M3, and M4 were 11.63 mm, 9.24 mm, and 12.06 mm, respectively. Additionally, the RMSE from M1 was relatively large in Test 2 (sit–to–stand) and Test 3 (full squat) compared to other tests, indicating that the S2J vector was significantly deformed from the initial S2J vector in these test motions. Whereas M1 does not reflect the deformation of the S2J vector based on STAs because it used constant vectors, M2–M4 used time-varying vectors, which can mitigate the effects of STAs. Compared with M2 and M3, which determine the S2J vectors from $f^{*}(x_{J,t})$ and $f^{*}(x_{J,1,t},x_{J,2,t})$, respectively, in the proposed method, M3 demonstrated higher estimation accuracy in most tests, although it differed depending on the test and joint. This indicates that, in terms of compensation of STA effects using the time-variation of S2J vectors, performance using two input variables is superior to that using a single variable. M4, which used estimated orientations (Rdpest) from IMMU signals, involved the orientation estimation errors listed in Table 3. Consequently, it resulted in larger estimation errors of relative position compared with M3 using the same approach.

As shown in Figure 6 and Figure 7, M1 showed a larger estimation error compared with the other methods. For Test 1 (knee lift), the estimation error from M1 significantly increased when the magnitude of the x-axis component of the relative position vector was the smallest. It should be noted that this moment is when the knee is maximally lifted. The estimation error from M1 was caused by only the deformation of the S2J vector, which increased as the S2J vector deformed differently from the predetermined constant vector. Therefore, it can be inferred that the S2J vectors were variated by the deformation of the lower body segments during knee lift. Similarly, in Test 3 (full squat), the estimation accuracy of M1 deteriorated significantly during the descent of a squat.

Meanwhile, the estimated relative positions from M2–M4 using the time-varying S2J vectors were relatively close to the truth reference and were not significantly affected by the body’s movements compared with M1, even in Tests 1 and 3 where the estimation error of M1 greatly increased. As M1–M3 differed only in terms of the S2J vectors because they involved the application of Rdpref in Equation (2), it was discovered that determining the S2J vector as a time-varying vector effectively compensated for the effects of body deformation. Furthermore, the STA compensation effects according to the application of the time-varying S2J vector can be clearly confirmed in the pelvis–to–foot result.

The estimation results from M4 were similar to those from M3 because the only difference between M3 and M4 was the orientations applied to Equation (2). It is noteworthy that the orientation in the proposed method was used not only to determine the relative position in Equation (2) but also to extract the joint angles ($x_{J,1,t}$ and $x_{J,2,t}$) for determining the S2J vectors. Hence, the accurate orientation estimation played a critical role in the estimation of relative position. Despite this performance degradation, M4 demonstrated a higher estimation accuracy than M1, indicating that the effects of body deformation can be sufficiently compensated using the estimated orientation.

**Figure 8:**
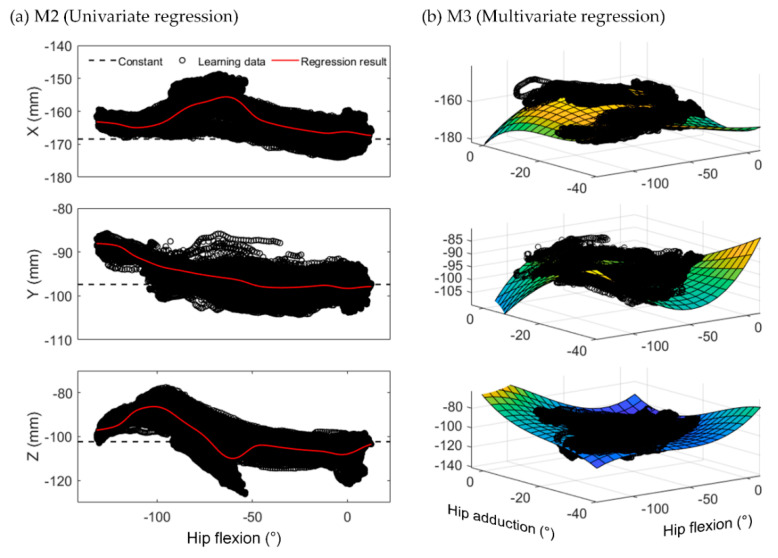
Regression results of S2J vectors for pelvis–to–hip from (**a**) M2 and (**b**) M3.

Again, M2 set a single variable as an input to the function for the S2J vector, whereas M3 set two variables. Therefore, we presumed that M3 may more precisely consider the effects of STAs. Figure 8 shows the regression results of the S2J vector for pelvis–to–hip (sPPH) from the two approaches. Both approaches derived the functions from the distribution of S2J vectors with respect to STA-related variables based on the learning data. Consequently, the regression results from M2 and M3 can be expressed as a one-dimensional nonlinear function and a curved surface, respectively. Because M3 used two variables to determine the S2J vector from the derived function, we assumed that it compensates for the effects of STAs more effectively. However, as shown in Table 2, M3 did not necessarily yield a higher estimation accuracy. For example, for Test 2 of Subject 1, the RMSE of M3 was higher than that of M2 in all relative positions except for shank–to–foot. Furthermore, for the pelvis–to–thigh motion of Test 4 from Subject 2, the error reductions of M2 and M3 relative to M1 were only 0.85 mm and 0.53 mm, respectively. These results were obtained because the proposed method mimicked only the average behavior of the S2J vectors for the STA-related variables, as shown in Figure 8. In other words, the proposed method exhibited uncertainties owing to the deviation of the S2J vector from the regression result.

**Figure 9:**
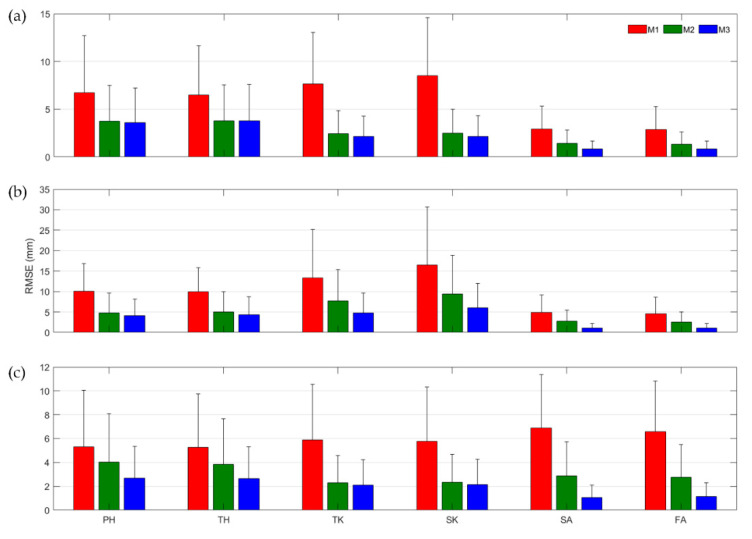
Averaged RMSE and the standard deviation of S2J vectors for learning data from (**a**–**c**) Subject 1–3: PH (pelvis–to–hip), TH (thigh–to–hip), TK (thigh–to–knee), SK (shank–to–knee), SA (shank–to–ankle), and FA (foot–to–ankle).

Figure 9 shows the averaged RMSE and the standard deviation of the constant (M1) and estimated S2J vectors obtained from M2 and M3 with respect to the reference S2J vectors of the learning data. For M2 and M3, because the time-varying S2J vectors were estimated from the learning data, the RMSEs of M2 and M3 were smaller than those of M1, which used the constant vector. However, the estimation error of the S2J vector was caused by uncertainty of the proposed method, which affected the relative position estimation. The large estimation errors and deviations of the S2J vectors were likely to result in inaccurately estimated relative positions. Nevertheless, all results from M2 and M3 except thigh/shank–to–knee for Subject 2 indicated RMSE values of less than 5 mm, indicating that the STA-related variables used in M2 and M3 exhibited some uncertainties but were correlated with the deformation of the S2J vector.

The uncertainties of the proposed method were associated with the complexity of the STAs, which varied with the motion, joint type, and physical characteristics of the subject (e.g., length of segment, muscle, and bone). Hence, it is impossible to thoroughly consider the effects of highly complex STAs using only a few variables. Nevertheless, it was confirmed that the application of the joint angles as the input variables for estimating the time-varying S2J vectors may effectively deal with the STA issue. Also, in case of the upper body, although not discussed in this paper, the joints exhibit ab/adduction and internal/external rotation in addition to flexion/extension. Accordingly, several angles that can consider the variation of S2J vectors more comprehensively should be selected to achieve precise relative position estimations.

Experimental results show that the proposed method may consider the effects of STAs in the relative position estimation, but there are still some limitations in practical application. In the proposed method, the estimation accuracy was highly correlated with the deviation of the motion from the mean represented by the derived function. Again, the proposed method took the average behavior of the S2J vectors for the STA-related variables. In this study, the functions outputting the S2J vectors were derived based on the limited number of specific motions (i.e., four types of tests). A higher number of motions represented by one regression model may decrease the estimation accuracy. One solution to this issue is to subdivide the function outputting the S2J vectors for each specific motion.

With regard to the sensor attachment, since the S2J vectors and joint angles were determined based on the fixed sensor attachment location/orientation, the estimation performance of the proposed method was sensitive to the sensor attachment state. Therefore, firm attachment of the sensor to segments is critical to limit the vibration or misalignment of the attached sensor [36].

## 4. Conclusions

A method was proposed to determine time-varying S2J vectors to compensate for the effects of STAs in IMMU-based relative positions between lower body segments. The proposed method expressed the S2J vector as a function of STA-related variables, which were highly correlated with the deformation of the S2J vectors owing to STAs, through regression. In this study, it was assumed that the joint angle was highly correlated with the deformation of the S2J vector, and two approaches were used, i.e., M2 and M3, which used one and two joint angles, respectively, as STA-related variables. The validation results showed that the proposed method successfully compensated for the effects of STAs in relative position estimation. In addition, it was confirmed that the compensation effect of the STA could be improved by using two joint angles rather than a single joint angle as the input variable for determining the S2J vector.

As a main contribution of this paper, this study quantitatively investigated the improvement of estimation accuracy of the relative position by reflecting the effects of STA, which is novel in IMMU-based human motion tracking. Furthermore, it is expected that the proposed method may work for the estimation of joint kinetic variables such as joint torques through IMMU-based inverse dynamics. This is because the improved joint kinematic information via the proposed method may consequently improve the estimation of joint kinetics, which is one of our future works. Although the proposed method considered the effects of STAs using physical quantities correlated with the deformation of the S2J vector, uncertainties remained. These uncertainties differ based on the joint types, movements, physical characteristics, etc. Therefore, appropriate variables should be selected to consider the effects of STAs more comprehensively such that they can be mitigated. Finally, by providing STA-compensated relative positions between segments, the proposed method applied in a wearable motion tracking system can be useful in rehabilitation or sports sciences.

## Figures and Tables

**Table 1:** Predetermined constant S2J vectors for Subjects 1–3 (unit: cm).

	Subject 1			Subject 2			Subject 3		
	X	Y	Z	X	Y	Z	X	Y	Z
PH	−16.84	−9.74	−10.23	−19.44	−4.43	−7.66	−17.26	−8.18	−12.80
TH	19.24	−0.58	−10.19	13.80	0.89	−11.05	22.46	0.00	−12.50
TK	−24.21	0.76	−5.88	−32.25	−0.53	−4.95	−22.59	2.05	−7.16
SK	23.08	−4.92	−9.33	16.01	−4.05	−5.03	19.84	−1.73	−10.91
SA	−18.22	−0.33	−4.00	−23.41	−3.43	−3.88	−23.79	−2.65	−4.14
FA	5.07	1.54	−4.36	6.03	2.55	−3.66	8.00	−0.31	−4.74

PH (pelvis–to–hip), TH (thigh–to–hip), TK (thigh–to–knee), SK (shank–to–knee), SA (shank–to–ankle), FA (foot–to–ankle).

**Table 2:** Averaged root mean squared errors (RMSEs) of the 3D relative position estimation for Subjects 1–3 (S1–S3) (unit: mm).

	Pelvis–to–Thigh				Thigh–to–Shank				Shank–to–Foot				Pelvis–to–Foot			
Test 1	M1	M2	M3	M4	M1	M2	M3	M4	M1	M2	M3	M4	M1	M2	M3	M4
S1	13.31	7.76	5.79	7.66	14.73	5.81	4.73	7.10	4.62	2.81	1.66	3.63	28.70	11.06	7.87	13.97
S2	23.80	11.29	10.46	10.66	15.11	15.87	9.04	8.98	6.38	6.34	3.96	4.37	26.03	19.82	16.61	18.43
S3	13.61	10.62	8.67	11.52	11.20	4.29	3.76	5.68	12.78	7.84	3.15	4.54	28.49	16.17	10.63	18.61
Avg	16.91	9.89	8.31	9.95	13.68	8.66	5.84	7.25	7.93	5.66	2.92	4.18	27.74	15.68	11.70	17.00
Test 2	M1	M2	M3	M4	M1	M2	M3	M4	M1	M2	M3	M4	M1	M2	M3	M4
S1	14.63	6.42	9.72	12.09	15.53	3.70	3.89	5.64	5.91	3.54	1.27	1.39	33.22	8.46	8.90	15.32
S2	27.26	11.04	8.49	9.37	24.91	12.88	13.68	13.49	9.58	6.38	2.06	1.94	50.36	24.45	19.58	19.53
S3	10.62	7.23	5.21	6.01	12.71	4.04	4.50	5.70	14.65	5.58	1.98	2.63	28.68	10.46	7.64	12.98
Avg	17.50	8.23	7.81	9.16	17.72	6.87	7.36	8.28	10.05	5.17	1.77	1.99	37.42	14.46	12.04	15.94
Test 3	M1	M2	M3	M4	M1	M2	M3	M4	M1	M2	M3	M4	M1	M2	M3	M4
S1	11.00	5.22	4.47	5.18	15.39	3.73	3.67	5.56	7.00	2.07	1.75	2.18	27.31	6.89	5.62	9.24
S2	12.09	9.90	8.34	8.57	35.43	14.62	12.81	12.88	12.96	4.21	1.90	2.11	47.44	21.89	17.88	20.37
S3	10.03	6.24	5.73	8.03	12.13	4.30	4.35	6.34	13.59	3.54	1.90	2.59	27.53	8.26	8.31	13.02
Avg	11.04	7.12	6.18	7.26	20.98	7.55	6.94	8.26	11.18	3.27	1.85	2.29	34.09	12.35	10.60	14.21
Test 4	M1	M2	M3	M4	M1	M2	M3	M4	M1	M2	M3	M4	M1	M2	M3	M4
S1	10.20	7.56	5.81	6.23	13.29	4.61	4.01	4.09	5.47	2.74	2.23	3.01	23.23	11.17	8.85	8.55
S2	11.05	10.20	10.52	11.66	27.56	14.18	7.99	8.94	8.39	4.25	1.73	2.02	34.50	22.61	15.35	21.07
S3	8.01	6.88	4.03	4.56	7.05	6.79	6.33	6.70	9.99	7.59	5.74	6.51	20.32	13.21	11.45	9.86
Avg	9.75	8.21	6.79	7.48	15.97	8.53	6.11	6.58	7.95	4.86	3.23	3.85	26.02	15.66	11.88	13.16

Test 1 (knee lift), Test 2 (sit–to–stand), Test 3 (full squat), Test 4 (half squat).

**Table 3:** Averaged RMSEs of the 3D joint angle estimation of each test (unit: °).

	Subject 1			Subject 2			Subject 3		
	Hip	Knee	Ankle	Hip	Knee	Ankle	Hip	Knee	Ankle
Test 1	1.83	1.41	2.32	1.58	1.64	1.14	1.51	1.44	1.55
Test 2	1.29	1.04	1.27	0.82	0.78	0.61	0.87	1.12	1.00
Test 3	0.89	1.20	1.38	0.63	1.07	0.68	1.22	1.09	1.22
Test 4	0.72	1.07	1.34	1.03	1.11	0.93	0.78	1.11	1.20

## Data Availability

The data presented in this study are available on request from the corresponding author.

## References

[1] Sabatini A.M. (2010). Kalman-filter-based orientation determination using inertial/magnetic sensors: Observability analysis and performance evaluation. Sensors.

[2] Lee J.K., Park E.J. (2009). Minimum-order Kalman filter with vector selector for accurate estimation of human body orientation. IEEE Trans. Robot..

[3] Ligorio G., Sabatini A.M. A linear kalman filtering-based approach for 3D orientation estimation from magnetic/inertial sensors. Proceedings of the 2015 IEEE International Conference on Multisensor Fusion and Integration for Intelligent Systems (MFI).

[4] Kang C.W., Kim H.J., Park C.G. (2016). A human motion tracking algorithm using adaptive EKF based on Markov chain. IEEE Sens. J..

[5] Lee J.K. (2019). A parallel attitude-heading Kalman filter without state-augmentation of model-based disturbance components. IEEE Trans. Instrum. Meas..

[6] Lotfi B., Huang L. (2016). An approach for velocity and position estimation through acceleration measurements. Measurement.

[7] Estrada A., Efimov D., Perruquetti W. Position and velocity estimation through acceleration measurements. Proceedings of the 19th World Congress of the International Federation of Automatic Control (IFAC 2014).

[8] Llorach G., Evans A., Agenjo J., Blat J. Position estimation with a low-cost inertial measurement unit. Proceedings of the 2014 9th Iberian Conference on Information Systems and Technologies (CISTI).

[9] Thong Y.K., Woolfson M.S., Crowe J.A., Hayes-Gill B.R., Jones D.A. (2004). Numerical double integration of acceleration measurements in noise. Measurement.

[10] Gilbert H.B., Celik O., O’Malley M.K. Long-term Double Integration of Acceleration for Position Sensing and Frequency Domain System Identification. Proceedings of the 2010 IEEE/ASME International Conference on Advanced Intelligent Mechatronics.

[11] Lee J.K., Park E.J. (2010). Quasi real-time gait event detection using shank-attached gyroscopes. Med. Biol. Eng. Comput..

[12] El-Gohary M., Pearson S., McNames J., Mancini M., Horak F., Mellone S., Chiari L. (2014). Continuous monitoring of turning in patients with movement disability. Sensors.

[13] Zhao H., Wang Z., Qiu S., Wang J., Xu F., Wang Z., Shen Y. (2019). Adaptive gait detection based on foot-mounted inertial sensors and multi-sensor fusion. Inf. Fusion.

[14] Dadashi F., Mariani B., Rochat S., Büla C.J., Santos-Eggimann B., Aminian K. (2014). Gait and foot clearance parameters obtained using shoe-worn inertial sensors in a large-population sample of older adults. Sensors.

[15] Alvarez J.C., Alvarez D., López A., González R.C. (2012). Pedestrian navigation based on a waist-worn inertial sensor. Sensors.

[16] Zizzo G., Ren L. (2017). Position tracking during human walking using an integrated wearable sensing system. Sensors.

[17] Lee M.S., Ju H., Song J.W., Park C.G. (2015). Kinematic model-based pedestrian dead reckoning for heading correction and lower body motion tracking. Sensors.

[18] Tjhai C., O’Keefe K. (2019). Using step size and lower limb segment orientation from multiple low-cost wearable inertial/magnetic sensors for pedestrian navigation. Sensors.

[19] Kortier H.G., Sluiter V.I., Roetenberg D., Veltink P.H. (2014). Assessment of hand kinematics using inertial and magnetic sensors. J. Neuroeng. Rehabil..

[20] Atrsaei A., Alarieh H. (2018). Human arm motion tracking by inertial/magnetic sensors using unscented Kalman filter and relative motion constraint. J. Intell. Robot. Syst..

[21] Zhou H., Hu H. (2009). Reducing drifts in the inertial measurements of wrist and elbow positions. IEEE Trans. Instrum. Meas..

[22] Schepers M., Giuberti M., Bellusci G. (2018). Xsens MVN: Consistent Tracking of Human Motion Using Inertial Sensing. Xsens Technol. B.V..

[23] Fasel B., Spörri J., Gilgien M., Boffi G., Chardonnens J., Müller E., Aminian K. (2016). Three-dimensional body and centre of mass kinematics in alpine ski racing using differential GNSS and inertial sensors. Remote Sens..

[24] Cameron J., Lasenby J. A Real-time Sequential Algorithm for Human Joint Localization. Proceedings of the ACM SIGGRAPH 2005 Posters.

[25] McGinnis R.S., Perkins N.C. (2013). Inertial sensor based method for identifying spherical joint center of rotation. J. Biomech..

[26] Seel T., Raisch J., Schauer T. (2014). IMU-based joint angle measurement for gait analysis. Sensors.

[27] Akbarshahi M., Schache A.G., Fernandez J.W., Baker R., Banks S., Pandy M.G. (2010). Non-invasive assessment of soft-tissue artifact and its effect on knee joint kinematics during functional activity. J. Biomech..

[28] Benoit D.L., Damsgaard M., Andersen M.S. (2015). Surface marker cluster translation, rotation, scaling and deformation: Their contribution to soft tissue artefact and impact on knee joint kinematics. J. Biomech..

[29] Grimpampi E., Camomilla V., Cereatti A., de Leva P., Cappozzo A. (2014). Metrics for describing soft-tissue artefact and its effect on pose, size, and shape of marker clusters. IEEE Trans. Biomed. Eng..

[30] Bonnet V., Richard V., Camomilla V., Venture G., Cappozzo A., Dumas R. (2017). Joint kinematics estimation using a multi-body kinematics optimisation and an extended Kalman filter and embedding a soft tissue artefact model. J. Biomech..

[31] Frick E., Rahmatalla S. (2018). Joint center estimation using single-frame optimization: Part 1: Numerical simulation. Sensors.

[32] García-de-Villa S., Jiménez-Martín A., García-Domínguez J.J. (2021). Novel IMU-based adaptive estimator of the center of rota-tion of joints for movement analysis. IEEE Trans. Instrum. Meas..

[33] Lee C.J., Lee J.K. (2020). Relative position estimation using Kalman filter based on inertial sensor signals considering soft tissue artifacts of human body segments. J. Sens. Sci. Technol..

[34] Nadaraya E.A. (1964). On estimating regression. Theory Probab. Appl..

[35] MATLAB Central File Exchange. https://www.mathworks.com/matlabcentral/fileexchange/34765-polyfitn.

[36] Stetter B.J., Ringhof S., Krafft F.C., Sell S., Stein T. (2019). Estimation of knee joint forces in sport movements using wearable sensors and machine learning. Sensors.

